# Prevalence of Abnormalities in Vestibular Function and Balance among HIV-Seropositive and HIV-Seronegative Women and Men

**DOI:** 10.1371/journal.pone.0038419

**Published:** 2012-05-31

**Authors:** Helen S. Cohen, Christopher Cox, Gayle Springer, Howard J. Hoffman, Mary A. Young, Joseph B. Margolick, Michael W. Plankey

**Affiliations:** 1 Bobby R. Alford Department of Otolaryngology – Head and Neck Surgery, Baylor College of Medicine, Houston, Texas, United States of America; 2 Department of Epidemiology, Johns Hopkins University, Bloomberg School of Public Health, Baltimore, Maryland, United States of America; 3 Epidemiology and Statistics Program, National Institute on Deafness and Other Communication Disorders, National Institutes of Health, Bethesda, Maryland, United States of America; 4 Division of Infectious Diseases, Department of Medicine, Georgetown University Medical Center, Washington, D. C., United States of America; 5 Department of Molecular Microbiology and Immunology, Johns Hopkins University, Bloomberg School of Public Health, Baltimore, Maryland, United States of America; University of Pittsburgh, United States of America

## Abstract

**Background:**

Most HIV-seropositive subjects in western countries receive highly active antiretroviral therapy (HAART). Although many aspects of their health have been studied, little is known about their vestibular and balance function. The goals of this study were to determine the prevalences of vestibular and balance impairments among HIV-seropositive and comparable seronegative men and women and to determine if those groups differed.

**Methods:**

Standard screening tests of vestibular and balance function, including head thrusts, Dix-Hallpike maneuvers, and Romberg balance tests on compliant foam were performed during semiannual study visits of participants who were enrolled in the Baltimore and Washington, D. C. sites of the Multicenter AIDS Cohort Study and the Women's Interagency HIV Study.

**Results:**

No significant differences by HIV status were found on most tests, but HIV-seropositive subjects who were using HAART had a lower frequency of abnormal Dix-Hallpike nystagmus than HIV-seronegative subjects. A significant number of nonclassical Dix-Hallpike responses were found. Age was associated with Romberg scores on foam with eyes closed. Sex was not associated with any of the test scores.

**Conclusion:**

These findings suggest that HAART-treated HIV infection has no harmful association with vestibular function in community-dwelling, ambulatory men and women. The association with age was expected, but the lack of association with sex was unexpected. The presence of nonclassical Dix-Hallpike responses might be consistent with central nervous system lesions.

## Introduction

A few studies have reported that HIV infection is associated with vestibular impairment, especially among individuals with advanced HIV disease [1], [2]. Those studies were limited by being performed before the availability of highly active combination antiretroviral drug regimens [3], [4], [5], [6], [7]; small sample sizes [2], [5], [6], [8], [9]; use of participants with progressive HIV disease who also had chronic, non-vertiginous dizziness [2], or who had been given ototoxic medications [5], [6]; or use of only indirect tests of vestibular function [5], [10].

In one report patients with advanced HIV disease had more signs of central vestibular impairments than patients with less advanced disease but the level of HIV disease was not related to the incidence of peripheral vestibular impairments [11]. In another report HIV-seropositive (HIV+) subjects without neurologic symptoms but with increased latencies of auditory brainstem responses, decreased smooth pursuit, and decreased saccadic eye movements, had normal peripheral vestibular function as indicated by bi-thermal caloric test scores [8]. Those test results suggested that subjects had central neurologic involvement. Small post-mortem studies have shown histopathologic changes in the vestibular labyrinth among patients who died from AIDS complications [12]. Therefore, the relationship between HIV infection and vestibular function among community-dwelling, ambulatory adults remains unclear. The virus may affect the vestibular system directly and the central vestibular pathways indirectly via opportunistic infections that may cause secondary influences [13].

The vestibular system controls the vestibulo-ocular reflex (VOR) through specific brainstem nuclei that control signals to the extra-ocular muscles. Therefore, screening and diagnostic tests of the VOR assess vestibular function directly. Two widely used screening tests of the VOR are the head thrust test, which detects abnormal function of the horizontal semicircular canal or superior vestibular nerve [14], [15], [16], and the Dix-Hallpike maneuver [17], which is the standard test for benign paroxysmal positional vertigo (BPPV) of the posterior semicircular canal. Balance testing is an indirect measure of vestibular function because vestibular input to the spinal cord via the vestibulospinal tracts is one of several contributions to balance. Variations of the Romberg test are widely used for diagnostic tests on computerized force platforms [18] and for screening on non-computerized compliant foam [19], [20], [21].

In the present study we used the head thrust test, Dix-Hallpike maneuver, and Romberg tests to screen HIV+ and HIV-seronegative (HIV−) men and women who were participating in two longitudinal cohort studies. The goal was to determine if HIV+ people treated with HAART had a higher prevalence of vestibular disorders than HIV− people of comparable age, sex and racial/ethnic backgrounds.

## Methods

### Ethics Statement

The Institutional Review Boards for Johns Hopkins University, Georgetown University, Whitman-Walker Health and Baylor College of Medicine approved this study. All participants signed informed consent prior to participating.

### Study Sample

Participants from the Baltimore-Washington, DC site of the Multicenter AIDS Cohort Study (MACS) and the Washington, DC site of the Women's Interagency HIV Study (WIHS) were enrolled in this study. The MACS and WIHS are ongoing longitudinal studies of the natural and treated histories of HIV infection in men and women, respectively; the study designs have been described elsewhere [22], [23], [24]. Only methods relevant to the present study are presented here.

Every six months MACS and WIHS participants complete comprehensive physical examinations and provide blood specimens for CD4+ and CD8+ T cell counts by standardized flow cytometry [25] and for plasma HIV RNA determination. In the MACS, HIV RNA was measured using the COBAS Ultrasensitive Amplicor HIV-1 monitor assay (Roche Molecular Systems, Branchburg, NJ), sensitive to 50 copies HIV RNA/mL. In the WIHS, HIV RNA was measured using COBAS AmpliPrep/COBAS TaqMan HIV–1 Test (Roche Molecular Systems, Branchburg, NJ), sensitive to 48 copies HIV RNA/mL. Participants also complete interviewer-administered questionnaires, including questions about medical conditions, medical treatments, sexual behavior, illegal drug use, and cigarette and alcohol consumption since the previous visit. Self-reported use of antiretroviral medications is summarized at each visit to define whether participants have ever used mono- or combination antiretroviral therapy [26], or highly active antiretroviral therapy (HAART) [27].

Participants were excluded from the present study if they had had spinal injury, radiation to the neck or spine, vestibular impairment, or use of narcotics, antihistamines or sedatives within 48 hours of testing because those medications have inhibitory effects on the vestibular system.

### Testing Paradigm

#### Head thrust test

To perform the head thrust test [15] the examiner sat in front of the subject, instructed the subject to stare at the examiner's nose, held the subject's head with both hands, and rapidly turned the head approximately 10° to the left or right. The expected eye movement was a smooth, slow rotation of the eye away from the direction of the stimulus, representing the VOR. The test was rated as abnormal if the observer saw a catch-up saccade, i.e. a rapid, jumping motion of the eye that occurs if the VOR does not compensate well for the head movement. An abnormal response is consistent with impaired horizontal semicircular canal or superior vestibular nerve function.

#### Other eye movement tests

During the other eye movement tests, i.e., spontaneous nystagmus, gaze-evoked nystagmus, and Dix-Hallpike maneuvers, subjects wore goggles fitted with a monocular infra-red video-oculography camera (VOG) that recorded eye movements while the eyes were open in darkness to avoid the influence of vision on the responses. For all eye movement tests performed with VOG the outcome variable was nystagmus, i.e., a repetitive eye movement with alternating slow and fast phases.

Spontaneous nystagmus and gaze-evoked nystagmus were tested first, for quality control. Spontaneous nystagmus that occurs during quiet sitting in darkness indicates either an acute vestibular impairment or a central neurologic lesion. Eye movements were recorded while the subject sat quietly, in darkness, for at least 10 seconds. The presence of spontaneous nystagmus was considered abnormal. Gaze-evoked nystagmus, i.e. nystagmus elicited by lateral gaze, indicates either advanced age or a lesion in the central oculomotor pathway. Gaze-evoked nystagmus was recorded by asking the subject to look to the right or left by moving the eyes without moving the head. The presence of more than 2 beats of nystagmus was rated as abnormal.

The Dix-Hallpike maneuver [17] was administered by turning the front of the participant's head approximately 45° toward the test side, and then rapidly tilting the individual backward from the long-sitting position into the supine-lying position on the treatment table, with the head hanging off the end of the table and pitched upward approximately 30° while supported by the examiner's hand [28]. The head is typically held still for up to 30 seconds to observe for the onset of nystagmus. If nystagmus occurred the examiner waited until it stopped before allowing the subject to sit up. VOG recordings were subsequently reviewed by one of three observers who were not otherwise involved in the study, and who were expert raters with 7 to 20 years of experience performing this kind of vestibular testing.

Nystagmus elicited by the Dix-Hallpike maneuver was considered abnormal and was designated as classical or nonclassical [28], [29]. A classical pattern of nystagmus is characterized by a delay of several seconds before the onset of eye movement, during which a few low amplitude beats are followed by a brief “burst” of high amplitude nystagmus and then a decline in amplitude over 3 to 60 seconds; the nystagmus has horizontal and torsional components with the quick phase beating toward the stimulus side, and a vertical component with the quick phase beating upward. Nystagmus which did not fit the classical pattern was defined as nonclassical. Classical nystagmus is pathognomonic for BPPV of the posterior semicircular canal. Nonclassical nystagmus is consistent with either BPPV of an anterior semicircular canal or a lesion in the central vestibular pathways. Anterior canal BPPV is rare.

#### Balance testing

Participants performed Romberg standing balance tests without shoes, but while wearing socks to standardize footwear [30], [31], [32], on the floor and on 10.2 cm thick, medium density, Sunmate® compliant foam (Dynamic Systems, Leicester, NC), with heels and toes together and arms crossed. The four test conditions were: eyes open on the floor, eyes closed on the floor, eyes open on the foam, and eyes closed on the foam (ECF). Of these conditions ECF is the most consistent with a vestibular impairment because vision was unavailable and kinesthetic and somatosensory input through the soles of the feet were not reliable sources of information, so the individual had to rely more on vestibular signals. Each trial of Romberg testing was performed for a maximum of 30 seconds. A participant who did not maintain balance for 30 seconds was given a second trial. Failure to maintain balance on the second trial for 30 seconds was considered abnormal [19].

### Statistical Analysis

Descriptive measures were sex; age at time of testing; race/ethnicity; CD4+ and CD8+ T cell counts and log_10_ plasma HIV RNA at the study visit closest to the date of the test; currently taking HAART at the time of testing (yes/no); ever having used monotherapy, combination antiretroviral therapy (ART), or HAART; nadir CD4+ T cell count; peak CD8+ T cell count; and T-cell counts at the study visit just prior to the time of testing.

Data on each outcome variable were coded as normal or abnormal using the definitions given above. The following outcome variables were analyzed using separate multivariable logistic regression models: Dix-Hallpike any nystagmus, Dix-Hallpike non-classical nystagmus, Romberg (ECF), and gaze-evoked nystagmus. The number of Dix-Hallpike classical nystagmus responses, head thrust responses, and spontaneous nystagmus were too small for multivariable modeling. Models were run using data from the combined HIV+ and HIV-participants and HIV+ participants only. The models using the combined population were adjusted for HIV status, sex, race/ethnicity (black or non-black), and age at testing. Additional covariates for models including only the HIV+ participations were: current CD4+ T cell count; current CD8+ T cell count; log_10_ plasma HIV RNA; ever monotherapy (yes or no); ever combination therapy (yes or no); and current HAART use (yes or no). In some analyses nadir CD4+ T cell count and peak CD8+ T cell count were included as covariates instead of the current T cell counts.

## Results

From March 2008 to September 2010 545 potential participants were screened for eligibility for the study prior to testing. Six people (1 HIV− woman; 2 HIV+ men, 3 HIV− men) declined to participate in the screening process. Also, for one or more of the following reasons 92 people were excluded: use of anti-nausea/vertigo medication, tranquilizer, sedatives, etc., in last 48 hours (n = 30); history of head or neck trauma including surgery or radiation to ear or neck (n = 23); use of a prosthetic leg or gait aid (n = 19); weight more than 136 kg (n = 18); lower limb injury or use of lower extremity orthosis (n = 15); history of spinal disease of neck (n = 8); paralysis of extremity on one side of body (n = 4); recent use of erectile dysfunction drugs and/or alcohol (n = 2); history of Meniere's disease (n = 1). After exclusions 447 participants (mean age = 51.3 years, SD = 10.3 years) completed balance and vestibular testing, including 294 men (65.8%) and 153 (34.2%) women.

**Table 1** shows the demographic characteristics of the participants. Fewer women were HIV− than HIV+, but the ages of all groups were similar. Most HIV+ participants i.e., 82% of women and 92% of men, had taken HAART at some point and most participants, i.e. 70% of women and 83% of men, were taking HAART at the time of data collection. Women and men differed on some measures. More women than men were black, and more women had been diagnosed with AIDS prior to the year preceding testing (36% vs. 17%). Virologic suppression to undetectable levels was lower among women than men (50% vs. 79%) but the mean CD4+ and CD8+ T cell counts were comparable.

**Table 1:** Enrollment characteristics of sample.

	HIV+			HIV−		
	Men	Women	All HIV+	Men	Women	All HIV−
Total, n	126	121	247	168	32	200
Age, mean (SD), yrs	52.0 (8.0)	45.6 (8.6)	48.9 (8.9)	56.7 (9.5)	40.9 (10.2)	54.2 (11.2)
Race, n (%)						
Non-black	58 (46.0)	26 (21.5)	84 (34.0)	134 (79.8)	9 (28.1)	143 (71.5)
Black	68 (54.0)	95 (78.5)	163 (66.0)	34 (20.2)	23 (71.9)	57 (28.5)
Ever AIDS, n (%)	22 (17.5)	44 (36.4)	66 (26.7)			
Ever mono, n (%)	37 (29.4)	47 (38.8)	84 (34.0)			
Ever combo, n (%)	59 (46.8)	70 (57.9)	129 (52.2)			
Ever HAART, n (%)	116 (92.1)	99 (81.8)	215 (87.0)			
Current HAART, n (%)	105 (83.3)	85 (70.3)	190 (76.9)			
Nadir CD4+ T cell count, mean (SD)	281.5 (174.1)	258.6 (183.0)	270.2 (178.5)			
Current CD4+ T cell count, mean (SD)	568.8 (277.0)	543.6 (291.7)	556.4 (284.0)			
Peak CD8+ T cell count, mean (SD)	1468.8 (699.8)	1371.7 (656.5)	1421.3 (679.3)			
Current CD8+ T count, mean (SD)	910.2 (403.4)	805.4 (361.9)	858.8 (386.4)			
Undetectable HIV RNA, n (%)	99 (78.6)	60 (49.6)	159 (64.4)			
Log_10_ HIV RNA, median (IQR)	3.94 (2.91, 4.45)	3.44 (2.67, 4.40)	3.50 (2.68, 4.42)			

Ever AIDS, ever diagnosed with AIDS; Ever mono, ever used monotherapy; Ever combo, ever used combination HAART and monotherapy; Ever HAART, ever used HAART. Undetectable HIV RNA, subjects with undetectable levels of HIV RNA; Log_10_ HIV RNA, log level of HIV RNA among HIV+ participants with detectable levels of RNA (n = 27 men and 61 women).

### Dix-Hallpike Maneuver

As each person has 2 vestibular labyrinths, one in each inner ear, six patterns of responses were possible: (actual numbers of subjects given in parentheses): negative bilaterally (249); negative unilaterally and positive, classical unilaterally (3); negative unilaterally and positive, nonclassical unilaterally (87); positive, classical bilaterally (3); positive, nonclassical bilaterally (68); positive, classical unilaterally and positive, nonclassical unilaterally (3). The notable findings are: a) most subjects were negative, bilaterally, b) few subjects had unilateral classical nystagmus, c) many more subjects had nonclassical nystagmus unilaterally or bilaterally.

As indicated in **Table 2** the overall prevalence of nystagmus was approximately 40%, slightly higher among women than men. The frequency was slightly, but not statistically significantly, higher among HIV+ compared to HIV− females. Multivariable analyses showed that HIV+ participants who were using HAART at the time of testing had a lower likelihood of having any nystagmus, which approached significance (OR = 0.47, 95% CI: 0.2, 1.03). The lower likelihood of having any nystagmus was associated with higher viral loads; that association approached statistical significance (OR = 0.74, CI = 0.52, 1.05).

**Table 2:** Frequency distribution of abnormal testing outcomes.

	HIV+, n (%)			HIV−, n(%)		
	Men	Women	All HIV+	Men	Women	All HIV−
Total	126	121	247	168	32	200
Dix-Hallpike any nystagmus*	44 (37.3)	49 (44.1)	93 (40.6)	58 (37.2)	13 (40.6)	71 (37.8)
Dix-Hallpike classical nystagmus	1 (0.9)	1 (0.9)	2 (0.9)	3 (1.9)	4 (12.5)	7 (3.7)
Dix-Hallpike nonclassical nystagmus	43 (36.4)	48 (43.2)	91 (39.7)	55 (35.3)	9 (28.1)	64 (34.0)
Head Thrust test	12 (9.8)	0	12 (5.0)	35 (21.1)	0	35 (17.7)
Romberg ECF	21 (17.7)	16 (13.6)	37 (15.6)	24 (14.7)	3 (9.4)	27 (13.9)
Spontaneous nystagmus	5 (4.3)	2 (1.8)	7 (3.1)	7 (4.4)	3 (9.4)	10 (5.2)
Gaze-evoked nystagmus	47 (39.8)	40 (36.4)	87 (38.2)	68 (42.8)	12 (37.5)	80 (41.9)

* Dix-Hallpike any nystagmus = Dix-Hallpike classical nystagmus+Dix-Hallpike nonclassical nystagmus.

The overall prevalence of classical nystagmus was higher among the HIV− participants compared to the HIV+ participants, but the groups did not differ significantly (3.7% vs 0.9%, p = 0.09). The prevalence was, however, significantly higher among the HIV− women compared to HIV+ women (12.5% vs 1%, p = 0.009) only. See **Table 2**.

The overall prevalence of nonclassical nystagmus was approximately 40%, similar to the value for any nystagmus. HIV+ participants currently using HAART had a lower likelihood of nonclassical nystagmus, which approached statistical significance (OR = 0.50, CI: 0.23, 1.11). Multivariable analysis showed that HIV+ participants who had never used HAART had a significantly higher risk of having nonclassical nystagmus (OR = 2.62, CI: 1.1, 6.23) compared to HIV+ subjects who had never used HAART.

No association was found between age, sex or race/ethnicity and any of the dependent variables in any of the models we tested. See **Tables 3** **and** **4** for data on any nystagmus and nonclassical nystagmus.

**Table 3:** Multivariable odds ratios (with 95% confidence intervals) from multiple logistic regression analysis for combined HIV+ and HIV−.

	Dix-Hallpike any nystagmus	Dix-Hallpike non-classical nystagmus	Romberg ECF	Gaze-evoked nystagmus
Age per 10 yrs increase	1.09 (0.87, 1.37)	1.14 (0.91, 1.43)	2.42 (1.73, 3.39)*	1.01 (0.81, 1.26)
Black vs. non-black	1.03 (0.65, 1.62)	1.06 (0.67, 1.68)	1.89 (0.97, 3.69)#	0.50 (0.32, 0.8)*
Female vs. male	1.31 (0.8, 2.15)	1.17 (0.71, 1.93)	1.1 (0.54, 2.24)	1.03 (0.62, 1.7)
HIV+ not HAART	1.41 (0.72, 2.78)	1.66 (0.84, 3.29)	1.93 (0.73, 5.07)	1.35 (0.67, 2.7)
HIV+ HAART	0.98 (0.62, 1.54)	1.17 (0.73, 1.85)	1.43 (0.73, 2.8)	1.04 (0.66, 1.63)

* Statistically significant at p<0.05.

# Statistically significant at p<0.09.

**Table 4:** Multivariable odds ratios (with 95% confidence intervals) from multiple logistic regression analysis for HIV+ only.

	Dix-Hallpike any nystagmus	Dix-Hallpike non-classical nystagmus	Romberg ECF	Gaze-evoked nystagmus
Age per 10 yrs increase	1.147 (0.8, 1.65)	1.1 (0.76, 1.58)	2.51 (1.49, 4.21)*	0.91 (0.63, 1.32)
Black vs. non-black	1.419 (0.77, 2.61)	1.32 (0.72, 2.43)	1.33 (0.56, 3.13)	0.47 (0.26, 0.87)*
Female vs. male	1.433 (0.75, 2.73)	1.43 (0.75, 2.72)	1.32 (0.54, 3.2)	1.06 (0.55, 2.05)
Current HAART	0.47 (0.21, 1.03)#	0.50 (0.23, 1.11)#	0.68 (0.23, 2.02)	0.45 (0.2, 1.03)#
Ever Combo	1.18 (0.63, 2.23)	1.21 (0.64, 2.27)	0.88 (0.37, 2.12)	1.02 (0.53, 1.96)
Ever Mono	0.75 (0.38, 1.49)	0.74 (0.37, 1.46)	0.70 (0.28, 1.73)	0.97 (0.48, 1.97)
Current CD4+ T cell count	1.00 (0.9, 1.12)	1.01 (0.9, 1.13)	0.90 (0.77, 1.05)	1.01 (0.9, 1.14)
Current CD8+ T cell count	1.01 (0.94, 1.09)	1.01 (0.94, 1.09)	1.06 (0.96, 1.16)	0.99 (0.92, 1.07)
Log_10_ HIV RNA	0.74 (0.52, 1.05)#	0.75 (0.53, 1.06)	0.86 (0.54, 1.36)	0.66 (0.45, 0.96)*

* Statistically significant at p<0.05.

# Statistically significant at p<0.09.

### Head Thrust Test and Spontaneous Nystagmus

No women and few men (9.8% of HIV+ and 21.1% of HIV−) had positive head thrust tests. Few subjects in each group had spontaneous nystagmus (3.1% HIV+ and 5.2% HIV−). See **Table 2**.

### Gaze-evoked Nystagmus

The overall prevalence of gaze-evoked nystagmus was approximately 39%, as indicated in **Table 2**. This prevalence was unexpectedly high. Being black appeared to be related to a lower risk of having gaze-evoked nystagmus in the combined HV+ and HIV− group (OR = 0.50, CI: 0.32, 0.80) and in the HIV+ group alone, (OR = 0.47, CI: 0.26, 0.87). Among HIV+ participants, the presence of gaze-evoked nystagmus was unrelated to CD4+ or CD8+ T cell counts, or use of antiretroviral medications. Multivariable analyses showed that in the HIV+ group current use of HAART was associated with a lower likelihood of having gaze-evoked nystagmus, which approached statistical significance (OR = 0.45, CI: 0.2, 1.03); the level of HIV RNA was inversely associated with the risk of having a positive response (OR = 0.66 per log increase, CI: 0.45, 0.96). The results were not related to age or sex. These findings must be interpreted with caution, however, due to the small number of subjects who had positive responses. See **Tables 3** **and** **4**.

### Romberg ECF

The overall prevalence of failure to stand during ECF for 30 seconds was 16% in men and 12.7% in women, slightly higher in HIV+ than HIV− subjects. See Table 2. The response was significantly related to subject age in multivariable models for both the combined sample (OR = 2.42, CI: 1.73, 3.39) and for the HIV+ sample (OR = 2.51, CI: 1.49, 4.21), but was not significantly associated with sex, race/ethnicity, CD4+ and CD8+ T cell counts, plasma HIV RNA, or use of antiretroviral medications. See **Tables 3** **and** **4**.

### Secondary Statistical Analyses

The results of additional models, including covariates such as nadir CD4+ T cell counts (replacing current CD4+ T cell count), peak CD8+ T cell counts (replacing current CD8+ T cell count) and ever using HAART (replacing current HAART use) did not differ significantly from the original models for head thrust, Romberg ECF or gaze-evoked nystagmus (data not shown). Ever having used HAART approached statistical significance with classical nystagmus and was significantly related to nonclassical nystagmus elicited by Dix-Hallpike maneuvers.

## Discussion

Peripheral vestibular impairment was not associated with HAART-treated HIV infection, as indicated by lack of significant difference between HIV+ and HIV− men and women on the head thrust test, Dix-Hallpike maneuver, and Romberg tests. These results differ from earlier findings [1], [11] that suggested a strong association of vestibular impairment among individuals who had more advanced HIV disease. The study by Hausler et al^1^. was performed before the advent of current antiretroviral medication regimens. Thus, the earlier findings do not apply to our sample of community-dwelling, HIV-infected individuals, most of whom were receiving HAART and had had at least some immune reconstitution.

In the general population BPPV is more common than other vestibular disorders, common in middle age, increases in frequency with age, and is approximately twice as prevalent in women as in men until old age [33], [34]. In that context several findings are noteworthy: 1) the lower likelihood of having positive responses among HIV+ subjects who used HAART; 2) the higher odds of having nonclassical nystagmus among subjects who had never used HAART; 3) the similar prevalences of classical Dix-Hallpike responses in HIV+ women and men, and 4) the lower prevalence of classical Dix-Hallpike responses among HIV+ women compared to HIV− women. The latter statement must be considered with caution because of the low number of positive, classical responses.

The low prevalence of classical responses in HIV+ and HIV− men is similar to the prevalence reported previously in HIV− men [33]. The unexpected trend toward association of lower frequency of any nystagmus with HAART use suggests a possible therapeutic relationship that requires further investigation. The reason for an apparent relationship between increased HIV RNA and lower likelihood of any nystagmus is unclear; it might be related to the relatively small number of participants with detectable HIV RNA [1], [11]. The reason for the approximately equal frequency of classical nystagmus among women and men in this study is unknown [33]. As we expected to see twice as many women as men with classical responses in HIV+ and HIV− groups, the lack of differences between men and women in the HIV+ group is surprising.

The higher percentage of nonclassical nystagmus elicited by the Dix-Hallpike maneuver may indicate BPPV of one of the anterior semicircular canals, which is less common than BPPV of the posterior semicircular canal [35], [36], although the exact frequency is unknown. Alternatively and more likely, because nonclassical nystagmus is generally considered as a central sign [37], these responses probably indicated impairments of the central vestibular pathways in the brain [28]. Therefore, the relationship between lower likelihood of nonclassical responses and current HAART use suggests an effect of HAART on central neurologic function.

The frequency of Dix-Hallpike responses should be considered in the context of the patterns of nystagmus. As expected, most participants had negative responses. Since most BPPV patients have unilaterally positive, classical responses and the prevalence of BPPV has been estimated as 1.6% [33] we expected that only a few subjects would have nonclassical responses. The surprisingly high prevalence of nonclassical responses might be consistent with impairments in oculomotor pathways.

The presence of gaze-evoked nystagmus among individuals who were not having acute attacks of labyrinthitis or vestibular neuronitis is consistent with involvement of central brain pathways related to oculomotor control rather than disorders of the peripheral vestibular system [38]. The finding of lower likelihood of gaze-evoked nystagmus in HIV+ subjects who are currently using HAART suggests a beneficial effect of HAART on central neurologic function.

The presence of a positive head thrust response is consistent with a clinically significant peripheral vestibular impairment. Men had relatively few positive responses but men in this age group are not known to have a high incidence of vestibular impairments. The finding that women had no positive head thrust responses was surprising because vestibular disorders are generally more prevalent among women [39]. This finding offers some support for the idea of a beneficial effect of HAART on the nervous system.

The Romberg ECF is a secondary, yet important, measure. Individuals with vestibular disorders are often impaired on performance of this test and it is widely used clinically. The lack of differences between HIV+ and HIV− groups on this test indicates that vestibularly mediated measures of balance were unaffected by HIV status. The relationship between age and failure on Romberg testing was expected. Age-related changes in vestibular and balance function are well known [40], [41], [42], [43], [44], [45]. Most participants were middle-aged. If the age ranges of the cohorts had been wider we might have found an even stronger influence of age on balance scores. These age-related balance changes did not appear to have functional correlations, as yet.

This study had several strengths. Most study participants were immunologically and virologically well-controlled [46], [47], none of whom had recently been diagnosed with any AIDS-defining conditions at the time of testing, and the cohort had similar numbers of male and female HIV+ individuals. Both cohorts are well-characterized with all of the testing procedures performed similarly. Participants were well known to the study staff and were not intimidated by testing. They were advised to seek health care if their test results showed positive responses and we established mechanisms for participants to obtain specialized medical care if they sought it. These humanitarian advantages to participants resulted in high rates of participation and reliable data.

The study had some limitations. This study was cross-sectional and therefore provides no data on the incidence of vestibular impairments. A longitudinal study would be necessary to address this question. Also, the VOG system did not allow calibration of saccades to exactly the same location in space; consequently measurement of gaze-evoked nystagmus was imprecise.

In general, our findings suggest that HIV infection treated with HAART is not associated with the development of peripheral vestibular impairments or premature aging of the vestibular labyrinth [46]. The effectiveness of HAART to prevent the progression of HIV is well established [47]. We have extended that general finding to show that HAART use was positively associated with a lower prevalence of vestibular impairment in community-dwelling, HIV-infected individuals. The findings from gaze-evoked nystagmus and nonclassical Dix-Hallpike responses might be associated with increased centrally mediated oculomotor deficits, perhaps consistent with mild neurologic disease found among HIV-infected individuals [8], [46].
